# Primary brain amyloidoma, both a neoplastic and a neurodegenerative disease: a case report

**DOI:** 10.1186/s12883-019-1274-x

**Published:** 2019-04-10

**Authors:** Mario Löhr, Almuth F. Kessler, Camelia-Maria Monoranu, Jens Grosche, Thomas Linsenmann, Ralf-Ingo Ernestus, Wolfgang Härtig

**Affiliations:** 1 Department of Neurosurgery, University Hospital of Wuerzburg, Josef-Schneider-Str. 11, 97080 Würzburg, Germany; 2 Department of Neuropathology, Institute of Pathology, University of Wuerzburg, Josef-Schneider-Str. 2, 97080 Würzburg, Germany; 3 Paul Flechsig Institute for Brain Research, University of Leipzig, Liebigstr. 19, 04103 Leipzig, Germany

**Keywords:** Amyloidoma, Neurooncology, Brain tumor, Neurodegenerative disease, Neurovascular unit

## Abstract

**Background:**

Scattered extracellular deposits of amyloid within the brain parenchyma can be found in a heterogeneous group of diseases. Its condensed accumulation in the white matter without evidence for systemic amyloidosis is known as primary brain amyloidoma (PBA). Although originally considered as a tumor-like lesion by its space-occupying effect, this condition displays also common hallmarks of a neurodegenerative disorder.

**Case presentation:**

A 50-year-old woman presented with a mild cognitive decline and seizures with a right temporal, irregular and contrast-enhancing mass on magnetic resonance imaging. Suspecting a high-grade glioma, the firm tumor was subtotally resected. Neuropathological examination showed no glioma, but distinct features of a neurodegenerative disorder. The lesion was composed of amyloid AL λ aggregating within the brain parenchyma as well as the adjacent vessels, partially obstructing the vascular lumina. Immunostaining confirmed a distinct perivascular inflammatory reaction. After removal of the PBA, mnestic impairments improved considerably, the clinical course and MRI-results are stable in the 8-year follow-up.

**Conclusion:**

Based on our histopathological findings, we propose to regard the clinicopathological entity of PBA as an overlap between a neoplastic and neurodegenerative disorder. Since the lesions are locally restricted, they might be amenable to surgery with the prospect of a definite cure.

## Background

Primary brain amyloidoma (PBA) is a rare tumor-like lesion characterized by a condensed deposition of amyloid within the brain parenchyma without evidence for systemic amyloidosis. PBA was originally reported by Saltykow in 1935 [1] followed by almost 40 additional cases with an average age of the patients at diagnosis of 49 (15–71) years [2]. However, the actual incidence of the condition is unknown. In almost all cases, the amyloid accumulations were found exclusively in the white matter of different supratentorial brain regions, predominantly in the frontal lobe and in proximity to the ventricular system [3, 4]. As neuroimaging features are multifaceted - except the disruption of the blood-brain-barrier (BBB) as the solely consistent finding - PBA was denominated a “chameleon-like lesion” due to the mimicking of different brain pathologies [3]. Apart from their neoplastic phenotype, as a slowly growing mass lesion, PBA’s are part of a complex group of progressive neurodegenerative disorders associated with an extracellular accumulation of fibrillar amyloid aggregates including, e.g., congophilic angiopathy and Alzheimer’s disease. Although composed of different precursor molecules, all cerebral amyloid deposition disorders entail common clinical symptoms, i.e., cognitive deficits, dementia, seizures, stroke, focal neurological signs, or a combination thereof [4].

### Case presentation

**Fig. 1:**
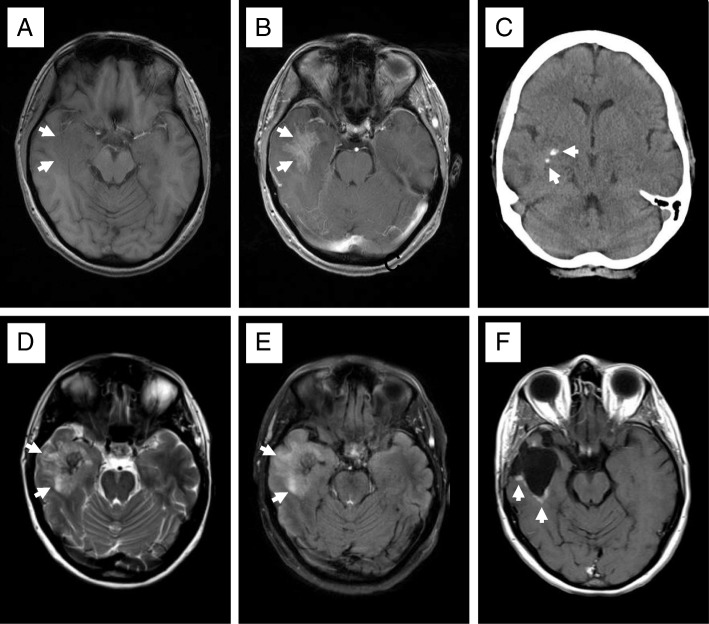
Neuroradiologic imaging pattern of primary brain amyloidoma. The T1-weighted MRI sequence shows a slightly hypointense right temporal mass (arrows in **a**), an ill-defined, moderate gadolinium enhancement (arrows in **b**), both compatible with malignant glioma. The CT displays small focal calcifications (arrows in **c**). The corresponding T2- and FLAIR MRI sequences displayed a hyperintense signal extending beyond the margins of the contrast enhancement indicating peritumoral edema or an infiltrating part of the mass (arrows in **d** and **e**). Almost 2 years following surgical removal of the PBA, the T1-weighted MRI sequence shows contrast enhancement at the resection margins (arrows in **f**) compatible with residual amyloidoma that remained unchanged during the 8-year follow-up

This 50-year-old female patient complained of a slow decline of her cognitive speed over several months and became symptomatic with generalized seizures. Her previous history revealed a myocardial infarction at the age of 42 years. Computerized tomography (CT) and magnetic resonance imaging (MRI) of the head showed a right temporal mass with focal calcifications, moderate perifocal edema and a reticulated contrast enhancement comprising neo- and allocortical regions (Fig. 1). Suspecting a high-grade glioma with an oligodendroglial component, the tumor was resected subtotally. Unexpectedly, the intraoperative finding was a firm, partially calcified and pink to greyish mass that was hypovascularized and poorly demarcated, so that no clear resection border could be defined intraoperatively.

Postoperatively, the patient recovered well, displayed a gradual cognitive improvement during the 24-month follow-up. She kept seizure-free under antiepileptic medication to date during the 8-year follow-up. MRI did not show any recurrence so far.

### Neuropathological findings

**Fig. 2:**
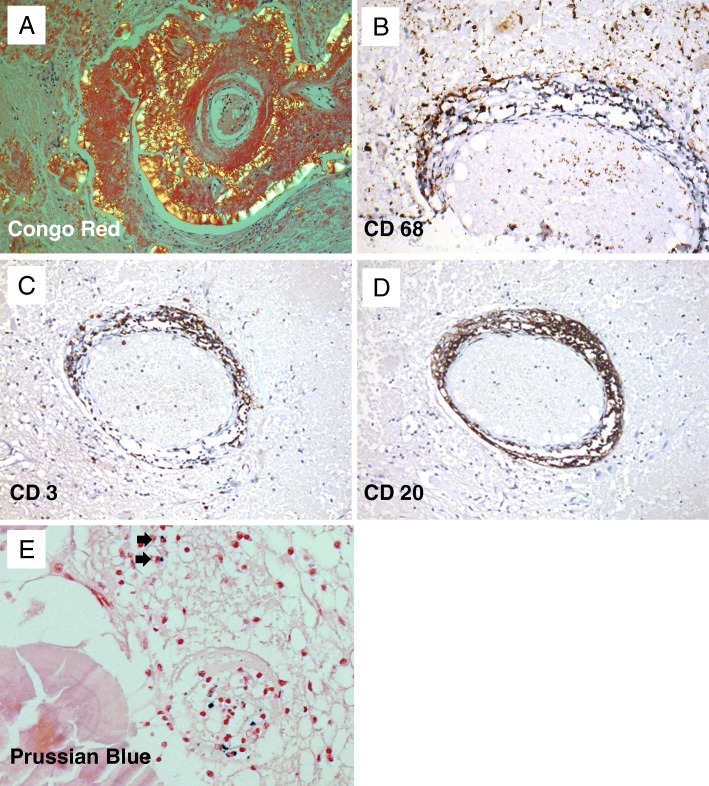
Histopathological and immunohistochemical characteristics of the cerebral amyloidoma. Large concentric sheets of Congo Red stained amyloid around an arteriole displaying the characteristic apple-green birefringence under polarized light and leading to an extraordinary thickening of the vessel wall (**a**), (original magnification × 25). Demonstration of the marked perivascular inflammatory reaction by visualizing macrophages (**b**) (CD68 immunostaining, original magnification × 200), T-lymphocytes (**c**), (CD3 immunostaining, original magnification × 100) and B-lymphocytes (**d**), (CD20 immunostaining, original magnification × 100). Hemosiderin-loaden macrophages indicating previous intraparenchymal microhemorrhage were encountered only very sporadically (arrows in **e**), (Prussian blue staining, original magnification × 50)

**Fig. 3:**
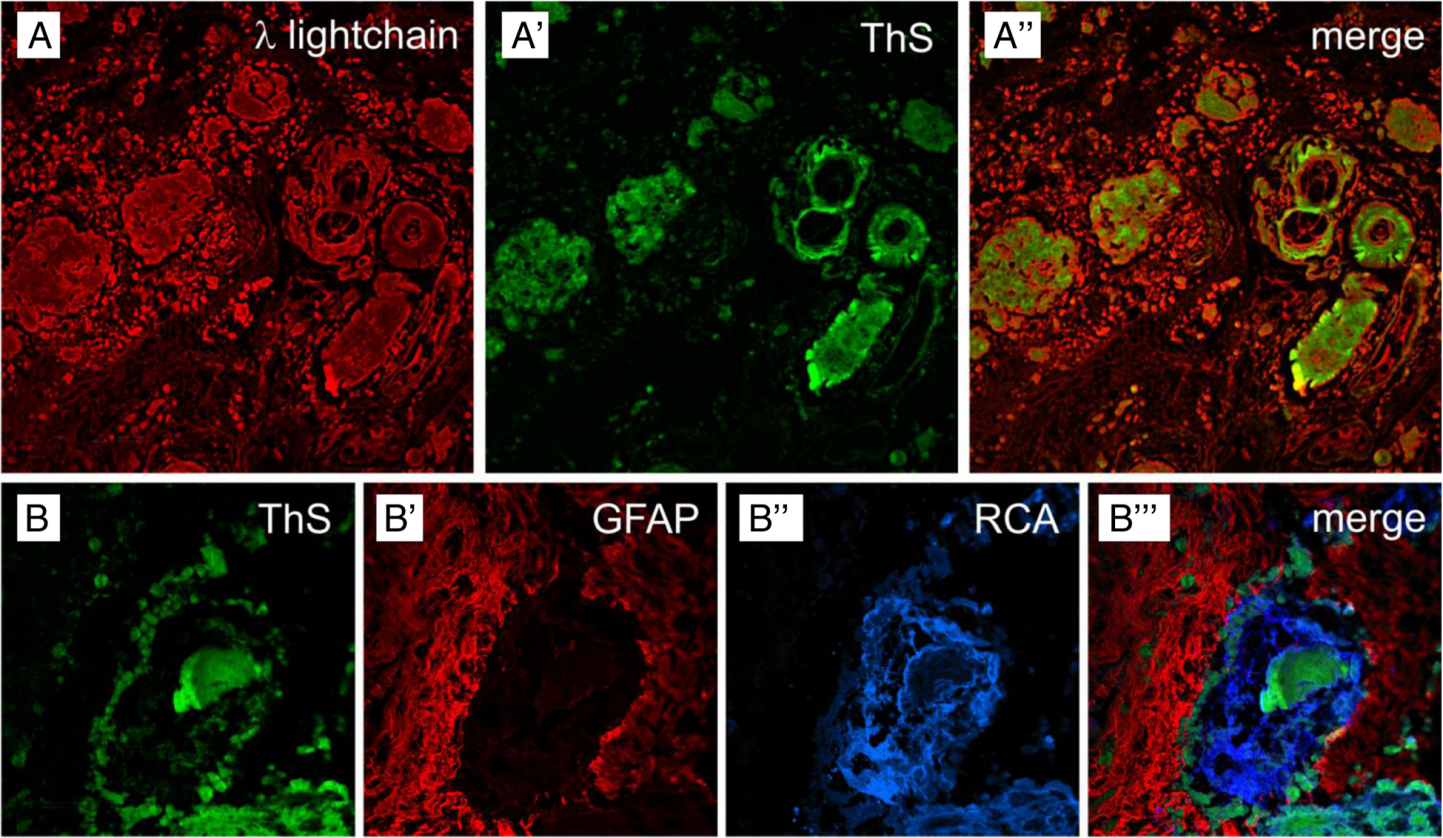
Confocal laser-scanning micrographs of the cerebral amyloidoma. Immunoreactivity for λ-light chain monomers (A) and thioflavin S (ThS)-stained amyloid in a paraffin section (A**´**). The overlay in (A**´´**) shows that the precursors are much more disseminated than their β-pleated self aggregates (original magnification × 60). Micrograph from a frozen section with thioflavin S-stained amyloid (B), combined with Cy3-immunolabeling of GFAP in perivascular astroglia (B**´**) and lectin-staining with *Ricinus communis* agglutinin (RCA) with Cy5, color-coded in blue (B**´´**). The allocation of markers becomes even clearer in the merged picture (B**´´´**), displaying amyloid deposits in the perivascular space, within the vessel wall and the vascular lumen (original magnification × 200)

Neuropathological examination showed no evidence of a glioma, but instead neuroectodermal tissue interspersed with scattered accumulations of periodic acid-Schiff (PAS)-positive, amorphous material located in the extracellular space that showed a distinct association to blood vessels where it assembled in a shell-like fashion. Congo Red staining clearly visualized extracellular amyloid deposits displaying apple-green birefringence under polarized light (Fig. 2a). Immunohistochemical analysis revealed aggregated λ-light chain (AL) amyloid with thioflavin S-stained β-pleated sheet conformation (Fig. 3A to A**´´**). In addition to the space-occupying effect of the widespread extracellular amyloid deposits, a marked dissociation of the vessel walls with subsequent stenosis (Fig. 1a) and even total obstruction of the vascular lumina became apparent (Fig. 3B to B´´´). Furthermore, the lesion was characterized by a distinct perivascular inflammatory reaction comprising of macrophages (Fig. 2b), microglia and T-lymphocytes (Fig. 2c), but also scattered B-lymphocytes and plasma cells (Fig. 2d). Despite the transmural inflammation and extensive amyloid deposition, there was no evidence of a resulting vessel fragility, since intraparenchymal hemorrhage was virtually absent (Fig. 2e). After identification of the tumor mass as a cerebral amyloidoma, systemic workup including rectal biopsy with negative results after Congo Red staining and echocardiography revealed no findings suspect for systemic amyloidosis, thus confirming the diagnosis of a PBA.

## Discussion and conclusions

PBA intriguingly share unequivocal features of two traditionally different nosological entities, i.e. cerebral neoplastic and neurodegenerative disorders. The localized aggregation of the amyloid in PBA suggests a synthesis of the insoluble protein at the site of the deposition rather than its leakage from the vessels [5]. As the amyloid of the cases reported almost exclusively was found to be of the light chain (AL) λ type, and in situ hybridization of scattered plasma cells squeezed between cerebral amyloid masses had shown a massive preponderance of λ producing cells, the concept to regard PBA as a low-grade monoclonal B-cell neoplasm capable of terminal differentiation had been proposed by Laeng et al. [6], but was later questioned by others [7, 8]. However, the main reason assigning PBA to cerebral neoplastic disorders is its progressive space-occupying effect, in combination with a disrupted BBB mimicking high-grade glioma [9], but rendering the lesions accessible for surgical treatment with a benign course thereafter [2, 3, 10]. As yet, there is only one report on a small recurrent lesion 15 years after complete excision [5].

Although PBA may be multiple and of variable sizes at presentation, the aggregates were always locally constricted and no dissemination within the brain was described so far, neither radiologically nor in necropsy cases [2, 5, 6, 11, 12]. Aside from PBA, brain-restricted light chain (AL) amyloid deposition is found in some different rare entities, presenting as single or multiple solid masses in solitary intracerebral plasmacytoma and primary intracerebral lymphoma with plasmacytic differentiation, and as disseminated lesions in multiple sclerosis with demyelination-associated amyloid aggregates, leptomeningeal vascular amyloidosis, and widespread subcortical vascular amyloidosis [4, 13, 14]. Since cerebral amyloid deposits can be associated with systemic amyloidosis, this has to be ruled by a thorough diagnostic workup [15, 16].

The main pathomorphological finding that underlines the neurodegenerative character of PBA are the vast peri- and intravascular amyloid deposits occurring in vessels adjacent to the parenchymal amyloid masses, probably reflecting a vascular clearance pathway of the neurotoxic amyloid by an active transport of the protein from the adventitial to the luminal side [5, 17]. In contrast to sporadic cerebral amyloid angiopathy (CAA), where the amyloid-ß deposits in the media and adventitia of cortical and leptomeningeal vessels lead to vascular fragility and rupture [18], the amyloid accumulations in PBA, though extensive, do not seem to increase the risk for intracerebral bleeding, since (micro)hemorrhages were virtually absent in the present case and are also not reported in the literature so far.

Due to the obstruction of parenchymal arterioles in PBA, local cerebral blood flow and the brain’s trophic support are severely impaired, disturbing BBB function and further aggravating the already diminished clearance mechanisms for the elimination of the insoluble protein aggregates [19, 20]. The perivascular space in PBA also houses a plethora of immune cells. Among the amyloid-induced perivascular inflammatory infiltrate [4], the abundant perivascular macrophages (PVMs) may act as scavenger cells to counteract the accumulation of amyloid that has been demonstrated in models of cerebral amyloid angiopathy [21]. Moreover, PVMs are a major source of reactive oxygen species. In animal models of Aß overproduction, the vascular oxidative stress by PVMs has been identified as a key effector for neurovascular and the ensuing cognitive dysfunction [22].

The final result of these self-amplifying effects is an enduring disturbance of the neurovascular unit in brain areas affected by AL-amyloid. Thus, a vicious circle of hypoxic/ischemic and neurodegenerative pathology arises, accelerated by the perivascular inflammation. This coexistence and mutual amplification of neurovascular dysfunction, inflammation and oxidative stress is detrimental to neuronal function and entails cognitive decline and focal neurological signs, as demonstrated for several forms of cerebral amyloidosis [4].

The histopathological findings of our case support the hypothesis, that PBA is a rare but unique clinicopathologic entity as it comprises distinct features of both, neoplastic and neurodegenerative disorders. Since the lesions are locally restricted and might be amenable to surgery, PBA has a prospect of definite cure in contrast to most other cerebral amyloid disorders.

## Abbreviations

AL: Amyloid light chain
BBB: Blood-brain-barrier
CAA: Cerebral amyloid angiopathy
CT: Computerized tomography
MRI: Magnetic resonance imaging
PBA: Primary brain amyloidoma
PVMs: Perivascular macrophages

## References

[1] Saltykow S (1935). Zur Frage des lokalen Amyloids der Hirngefäße. Virchows Arch.

[2] Heß K, Purrucker J, Hegenbart U, Brokinkel B, Berndt R, Keyvani K (2018). Cerebral amyloidoma is characterized by B-cell clonality and a stable clinical course. Brain Pathol.

[3] Fischer B, Palkovic S, Rickert C, Weckesser M, Wassmann HD (2007). Cerebral AL -λ amyloidoma: clinical and pathomorphological characteristics. Review of the literature and of a patient. Amyloid..

[4] Rostagno A, Holton JL, Lashley T, Revesz T, Ghiso J (2010). Cerebral amyloidosis: amyloid subunits, mutants and phenotypes. Cell Mol Life Sci.

[5] Westermark P (2012). Localized AL amyloidosis: a suicidal neoplasm?. Ups J Med Sci.

[6] Laeng RH, Altermatt HJ, Scheithauer BW, Zimmermann DR (1998). Amyloidomas of the nervous system. A monoclonal B-cell disorder with monotypic amyloid light chain λ amyloid production. Cancer..

[7] Foreid H, Barroso C, Evangelista T, Campos A, Pimentel J (2010). Intracerebral amyloidoma: case report and review of the literature. Clin Neuropathol.

[8] Tabatabai G, Baehring J, Hochberg FH (2005). Primary amyloidoma of the brain parenchyma. Arch Neurol.

[9] Ragel BT, Blumenthal DT, Browd SR, Salzman KL, Jensen RL (2006). Intracerebral amyloidoma can mimic high-grade glioma on magnetic resonance imaging and spectroscopy. Arch Neurol.

[10] Renard D, Campello C, Rigau V, de Champfleur N, Labauge P (2008). Primary brain amyloidoma. Long-term follow-up. Arch Neurol.

[11] Hori A, Kitamoto T, Tateishi J, Hann P, Friede RL (1988). Focal intracerebral accumulation of a novel type of amyloid protein. An early stage of cerebral amyloidoma?. Acta Neuropathol.

[12] Townsend JJ, Tomiyasu U, MacKay A, Wilson CB (1982). Central nervous system amyloid presenting as a mass lesion. J Neurosurg.

[13] Schröder R, Deckert M, Linke RP (2009). Novel isolated cerebral ALλ amyloid angiopathy with widespread subcortical distribution and leukoencephalopathy due to atypical monoclonal plasma cell proliferation, and terminal systemic gammopathy. J Neuropathol Exp Neurol.

[14] Pace AA, Lownes SE, Shivane A, Hilton DA, Weatherby SJ (2015). A tale of the unexpected: amyloidoma associated with intracerebral lymphoplasmacytic lymphoma. J Neurol Sci.

[15] Gertz MA, Lacy MQ, Dispenzieri A, Hayman SR (2005). Amyloidosis: diagnosis and management. Clin Lymphoma Myeloma.

[16] Wechalekar AD, Gillmore JD, Hawkins PN (2016). Systemic amyloidosis. Lancet..

[17] Iadecola C (2017). The neurovascular unit coming of age: a journey through neurovascular coupling in health and disease. Neuron..

[18] Salvarani C, Hunder GG, Morris JM, Brown RD, Christianson T, Giannini C (2013). Aß-related angiitis. Neurology..

[19] Hawkes CA, Härtig W, Kacza J, Schliebs R, Weller RO, Nicoll JA, Carare RO (2011). Perivascular drainage of solutes is impaired in the ageing mouse brain and in the presence of cerebral amyloid angiopathy. Acta Neuropathol.

[20] Iadecola C (2013). The pathobiology of vascular dementia. Neuron..

[21] Hawkes CA, McLaurin J (2009). Selective targeting of perivascular macrophages for clearance of beta-amyloid in cerebral amyloid angiopathy. Proc Natl Acad Sci U S A.

[22] Park L, Uekawa K, Garcia-Bonilla L, Kolzumi K, Murphy M, Pitstick R (2017). Brain perivascular macrophages initiate the neurovascular dysfunction of Alzheimer Aß peptides. Circ Res.

